# Preparation of Plaster Models

**Published:** 1856-10

**Authors:** 


PREPARATION OF PLASTER MODELS.—By immersing plaster models in a solution of carbonate of soda, their surface is converted into carbonate of lime. The surface is thus not only beautified, but rendered more permanent. It is a neat method of preparing models for preservation in cabinets. The solution should not contain any of the bi-carbonate.

